# Personal Verification/Identification via Analysis of the Peripheral ECG Leads: Influence of the Personal Health Status on the Accuracy

**DOI:** 10.1155/2015/135676

**Published:** 2015-10-19

**Authors:** Irena Jekova, Giovanni Bortolan

**Affiliations:** ^1^Institute of Biophysics and Biomedical Engineering, Bulgarian Academy of Sciences, Acad. G. Bonchev Street Bl 105, 1113 Sofia, Bulgaria; ^2^Institute of Neuroscience (IN), CNR, 35127 Padova, Italy

## Abstract

Traditional means for identity validation (PIN codes, passwords), and physiological and behavioral biometric characteristics (fingerprint, iris, and speech) are susceptible to hacker attacks and/or falsification. This paper presents a method for person verification/identification based on correlation of present-to-previous limb ECG leads: I (*r*_I_), II (*r*_II_), calculated from them first principal ECG component (*r*_PCA_), linear and nonlinear combinations between *r*_I_, *r*_II_, and *r*_PCA_. For the verification task, the one-to-one scenario is applied and threshold values for *r*_I_, *r*_II_, and *r*_PCA_ and their combinations are derived. The identification task supposes one-to-many scenario and the tested subject is identified according to the maximal correlation with a previously recorded ECG in a database. The population based ECG-ILSA database of 540 patients (147 healthy subjects, 175 patients with cardiac diseases, and 218 with hypertension) has been considered. In addition a common reference PTB dataset (14 healthy individuals) with short time interval between the two acquisitions has been taken into account. The results on ECG-ILSA database were satisfactory with healthy people, and there was not a significant decrease in nonhealthy patients, demonstrating the robustness of the proposed method. With PTB database, the method provides an identification accuracy of 92.9% and a verification sensitivity and specificity of 100% and 89.9%.

## 1. Introduction

The reliability of automatic person identification has become critical in our life, considering the necessary security for the cases of financial transactions, access control, travelling, and so forth. The traditional means for identity validation, such as PIN codes, passwords, and identity cards, are susceptible to hacker attacks and falsification. In the past few decades, identification based on physiological and behavioral biometric characteristics, such as fingerprint, iris, and speech, were proposed. However, these biometrics could be easily circumvented, for example, by using prosthetic finger or iris [1] or voice playback. Considering these drawbacks, recently the efforts are focused on the development of next generation of biometric characteristics that are internal to the human's body and therefore are robust to the above discussed attacks.

The analysis of the electrocardiogram (ECG) as a biometric tool was started about a decade ago and two general approaches could be distinguished: (i) methods that use measurements after detection of fiducial points and (ii) methods that analyze the overall morphology of the ECG.

The fiducial based approaches had been applied since the very beginning. One of the earliest studies that demonstrated the feasibility of ECG signals for biometrics [2] involved 12 uncorrelated clinical diagnosis features related to P, QRS, T amplitudes, and durations. The interpretation of the similarities/differences between individuals' heartbeats was performed by principal component analysis (PCA) score plots. The authors achieved classification rate of 100% using 10 of the features. Israel et al. [3] employed 15 temporal features describing the P-QRS-T segment which were fed into a set of discriminant functions for individual recognition. This group reported accuracy for the individual classification between 97% and 100%. In 2008, Wang et al. [4] introduced a two-step detection that incorporates temporal and amplitude measurements based on fiducial points detection and appearance based features that capture the patterns of the heartbeats. The authors achieved 100% subject identification based on this combined approach.

The methods incorporating time and amplitude characteristics of the heartbeats strongly rely on the correct localization of wave boundaries within the P-QRS-T segment. Current algorithms for ECG delineation are dedicated mainly to the medical applications where the detection of the approximate fiducial point positions is adequate for diagnostic purposes. In contrast, in order to reduce the rejection rate, perfect heartbeat synchronization is required for biometric purposes [5]. For that reason, fiducial independent approaches appeared after 2006. Great part of the proposed methods was based on calculation of correlation coefficients. Plataniotis et al. [6] proposed a method for personal identification applying autocorrelation (AC) of windowed ECG followed by discrete cosine transform (DCT) and reported 100% accuracy. Agrafioti and Hatzinakos [5] utilized the AC of 5 s ECG segments for biometric identification. The AC signals were processed by discriminant analysis and accuracy of 96.4% is reported. This work also presented an arrhythmia screening algorithm based on complexity measurement analysis which prevented considering ECG segments with ventricular ectopic beats. Poree et al. [7] reported 100% identification accuracy using the maximal correlation coefficient applied over 12-lead ECG. The accuracy dropped down to 91.4% when the method was applied over single ECG lead. Wübbeler et al. [8] formed a two-dimensional heart vector using limb leads ECGs, as well as its first and second temporal derivatives. The proposed identification relies on distance based approach and provides accuracy higher than 97%. Ye et al. [9] applied discrete wavelet transform (DWT) and independent component analysis (ICA) on ECG beats and obtained 136 features that were further reduced to 26 using PCA. The classification with SVM led to 99.6% accuracy. Recently, Zokaee and Faez [10] promoted a multimodal biometric system based on ECG and palm print analysis. They used Mel-frequency cepstrum coefficient (MFCC) approach to extract features of ECG biometrics and PCA to extract features from palm print. The accuracy provided by KNN classification was 94.7%. Sidek et al. [11] achieved personal recognition accuracy of 96.1% by feeding a normalized QRS complex into a Multilayer perceptron. Zhao et al. [1] reported a human ECG identification system based on ECG decomposition in a number of intrinsic mode functions combined with Welch spectral analysis for extraction of significant heartbeat features. PCA was used for feature space reduction. The classification with KNN method provided 95% identification accuracy.

Despite the reported high accuracy results and the reported evidences for ECG stability in different physiological conditions [3, 7, 11, 12] the validation of ECG for biometric identification requires more severe testing conditions and reduction to easily acquirable ECG leads in order to be adequate to the real situation and to be convenient and reliable for the person under identification process. In this respect, the cited papers have the following limitations:

1. Several studies use ECG recordings acquired in a very short temporal interval or in the same session [1, 3–5, 10, 11, 13] and this produces higher accuracy values. This fact was reported in a recent comparative analysis [14] over 20 authentication methods based on ECG analysis, where significant accuracy degradation was observed when training and testing data come from different sessions if compared to the case of single session.
2. The proposed methods are applied generally on healthy subjects. However, there are factors of pathologic nature that can severely influence ECG morphology and stability, such as transient or acute cardiac ischemia (manifested in ST-segment changes and sometimes in intra-QRS changes), hypertonia (high ECG voltage), ectopic beats, conductive anomalies causing sometimes intermittent bundle branch blocks, and paroxysmal atrial fibrillation. Many of these changes are of a long-lasting nature and could obviously influence the verification/identification accuracy.
3. Some studies are based on analysis over all standard 12 ECG leads [7] or on ECG acquired with electrodes placed on the chest [11]. However, the acquisition of the precordial leads is not realistic in many real situations or applications, and the analysis of reduced number of leads produces a reduction of the identification accuracy [7]. The acquisition of ECG for person identification is addressed by Chan et al. [15] and Lourenço et al. [13] who reported 95% and 94.3% accuracy using ECG leads acquired from palms and fingers, respectively.

The aim of this paper is to present, test, and validate a method for person verification and identification based on correlation using only the limb ECG leads. The method is developed and tested using a population based ECG database, in which the two recordings have been performed at an interval of 5 years. In addition, the database considers both healthy people and person with some cardiac disease or hypertension. The influence of the presence of nonhealthy patients in the validation phase is studied in detail.

## 2. Material and Methods

### 2.1. ECG Database

Two independent ECG databases have been used in this study for training and testing the proposed method: a population based database (ECG-ILSA) and a reference database present in many comparisons in literature (the PTB database).

#### 2.1.1. The ECG-ILSA Database

The ECG signals used for training and testing are taken from a computerized ECG-ILSA database, collected for the Italian Longitudinal Study on Aging Project [16–19]. A random sample of 5632 individuals aged from 65 to 84 years, living independently or in institutions, stratified by age and sex with an equal allocation strategy was identified on the demographic lists of the registry office of 8 Italian municipalities. They were followed up with an interval of 5 years in order to study and evaluate physiologic and pathologic modifications connected with aging. The computerized acquisition of ECG signals was performed in about 43% of the initial population.

This population based ECG-ILSA database consists of 2513 ECG signals in the first phase (*T*_1_) and 1352 ECG signals in the second phase (*T*_2_ = *T*_1_ + 5 years), and both ECGs are present in 901 patients. For this study, a subset of 540 subjects considered in a previous study [20] was selected. This group is consisting of 147 healthy subjects, 218 people with hypertension, and 175 with cardiac diseases. The healthy group is characterized by absence of cardiovascular and chronic pulmonary disease, no use of drugs that can influence the electrical cardiac activity, and no electrolyte imbalance. The cardiac group is characterized by 56 patients with single diagnosis of cardiovascular diseases, while, in the remaining group with multiple diagnoses, there are 51 patients with MI, 44 with ischemia, and 24 with both.

The ECG recordings are with duration of 10 s and they include the standard 12 leads, sampled at 500 Hz. In order to have a more robust validation procedure, considering the temporal variability of ECG signal and/or modifications in the pathologies, the learning phase was performed in the healthy group. For this purpose, a random subset of 98 ECGs from the healthy group at times *T*_1_ and *T*_2_ represents the training set. Consequently the remaining group of 49 healthy subjects and the entire cardiac and hypertension groups have been considered for the validation/testing procedure.

#### 2.1.2. The Reference PTB Database

We have used an additional test set, the PTB ECG database, which is a common reference database present in the literature for comparative results. The ECG signals are taken from the Physikalisch-Technische Bundesanstalt (PTB) database. The ECGs were collected from healthy volunteers and patients with different heart diseases. The database contains 549 records from 290 subjects, each one represented by one to five recordings, and it includes the conventional 12 leads together with the 3 Frank leads, sampled at 1000 Hz. The testing ECG set used in this study includes 14 healthy control subjects with multiple ECG recordings, for whom the first (*T*_1_) and the last (*T*_2_) ECG recordings have been considered. This dataset is characterized by a short time interval between the two acquisitions at *T*_1_ and *T*_2_: they were performed mainly in a temporal interval from hours (in half of patients) to some months. These ECG recordings have been used in literature as a reference for the evaluation of the methods for person verification/identification [4, 5].

### 2.2. Methods

Aiming at a practicable biometric system, the presented method operates over 10 s ECG segments and uses only the limb leads I and II. To minimize the negative effect of random noises the ECG signals were passed through

- high-pass filter with 0.64 Hz cutoff frequency to suppress baseline drift,
- low-pass filter with cutoff frequency 35 Hz to reduce muscle noise,
- a notch filter to eliminate power-line interference.

The filtered signals were subjected to QRS detection [21], providing the R-peak positions (QRS_index_). The QRS detection was based on comparison of a complex lead, representing the sum of the absolute values of the differentiated lead I and lead II with a combined adaptive threshold

(1) LeadQRSdet⁡i=absLeadIi−LeadIi−1+absLeadIIi−LeadIIi−1.

For the purposes of biometric recognition, an ECG from Set2 (2nd recording) is compared to the ECG in Set1 (1st recording) by applying the following procedures:

(1) Calculation of the mean RR interval of the tested ECG_Set2_ and the ECG_Set1_ involved in the current comparison (the smaller RR interval is further referred to as RR_min_).
(2) Opening of a window (QRS_index_ − RR_min_/3 to QRS_index_ + 2RR_min_/3) around the QRS complexes detected in ECG_Set1_ and ECG_Set2_.
(3) Application of principle component analysis (PCA) over the opened window, for derivation of combined information for the waveforms in leads I and II.
(4) Calculation of the correlation between each couple QRS_Set1_, QRS_Set2_, using the equation below:
  (2) rQRSSet2,QRSSet1=∑i=QRSindex−RRmin/3QRSindex+2RRmin/3QRSSet2iQRSSet1i∑i=QRSindex−RRmin/3QRSindex+2RRmin/3QRSSet2i2∑i=QRSindex−RRmin/3QRSindex+2RRmin/3QRSSet1i2.

The maximal correlation coefficient representing the best correlated couple QRS_Set1_, QRS_Set2_ is considered. The values of three independent correlation coefficients—*r*_I_ (for the heartbeats in lead I), *r*_II_ (lead II), and *r*_PCA_ (for the first principal component), two combined correlation coefficients (*r*_I_ + *r*_II_)/2, (*r*_I_ + *r*_II_ + *r*_PCA_)/3, and two nonlinear combinations between them representing the minimal value min(*r*_I_, *r*_II_, *r*_PCA_) and the maximal value max(*r*_I_, *r*_II_, *r*_PCA_) are further analyzed over the training dataset.

There are two typical scenarios for application of biometric recognition.

*(1) Person Verification.* The one-to-one scenario is applicable; that is, the ECG of the tested subject in *T*_2_ is compared to previously recorded ECG in *T*_1_ with known identity. If the maximal correlation is above a preset threshold value (CorrThr), it is accepted that both ECGs belong to one and the same person and the identity of the tested person is verified.

The accuracy for person verification over the training database is represented by sensitivity (Se_verification) and specificity (Sp_verification). Se_verification is calculated as the percentage of subjects for whom the assessed correlation coefficients and their combinations are above preset threshold values when their ECG signals in *T*_1_ and *T*_2_ are compared:

(3) Se_verification=100∑i=1NrQRSiT2,QRSiT1≥CorrThrN,

where *N* is the number of tested subjects.

Sp_verification is the percentage of cases for which the assessed correlation coefficients and their combinations are below the preset threshold values when comparing ECGs of different subjects:

(4) Sp_verification=100·∑i=1N∑j=1NrQRSiT2,QRSjT1<CorrThrNNtotal−1,i≠j,

where *N* is the number of tested subjects and *N*_total_ is the number of subjects in the database for comparison.

**Figure 1:**
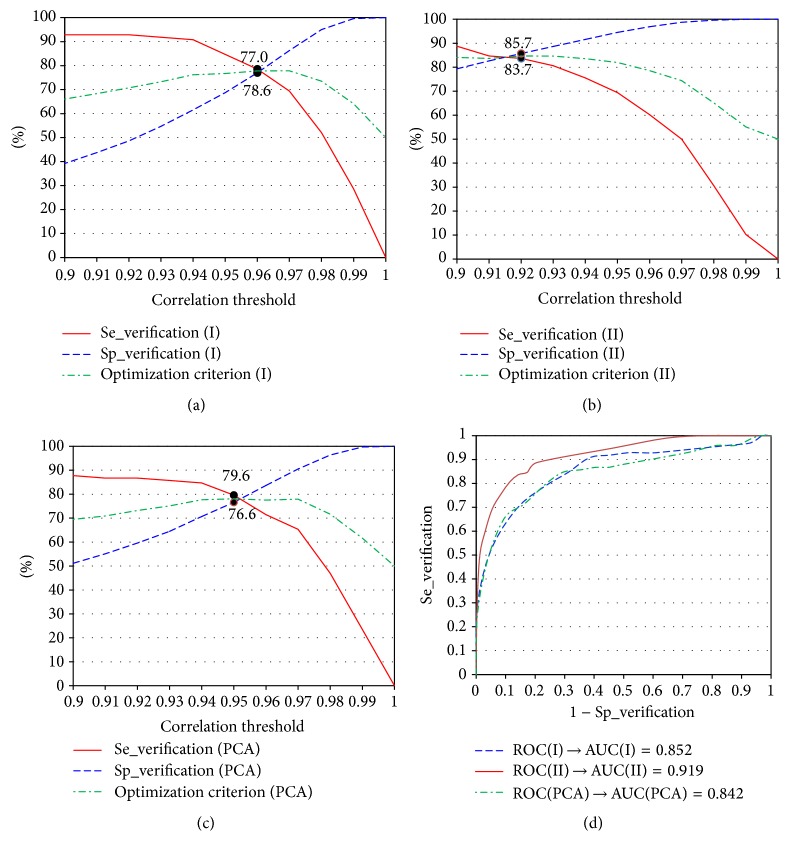
Influence of the threshold for *r*_I_, *r*_II_, and *r*_PCA_ on the person verification accuracy: (a) Se_verification (I) = *f*(*r*_I_); Sp_verification (I) = *f*(*r*_I_). (b) Se_verification (II) = *f*(*r*_II_); Sp_verification (II) = *f*(*r*_II_). (c) Se_verification (PCA) = *f*(*r*_PCA_); Sp_verification (PCA) = *f*(*r*_PCA_). (d) ROC curves and calculated AUC for classification based on *r*_I_, *r*_II_, and *r*_PCA_. The solid circles mark the optimal solutions.

**Figure 2:**
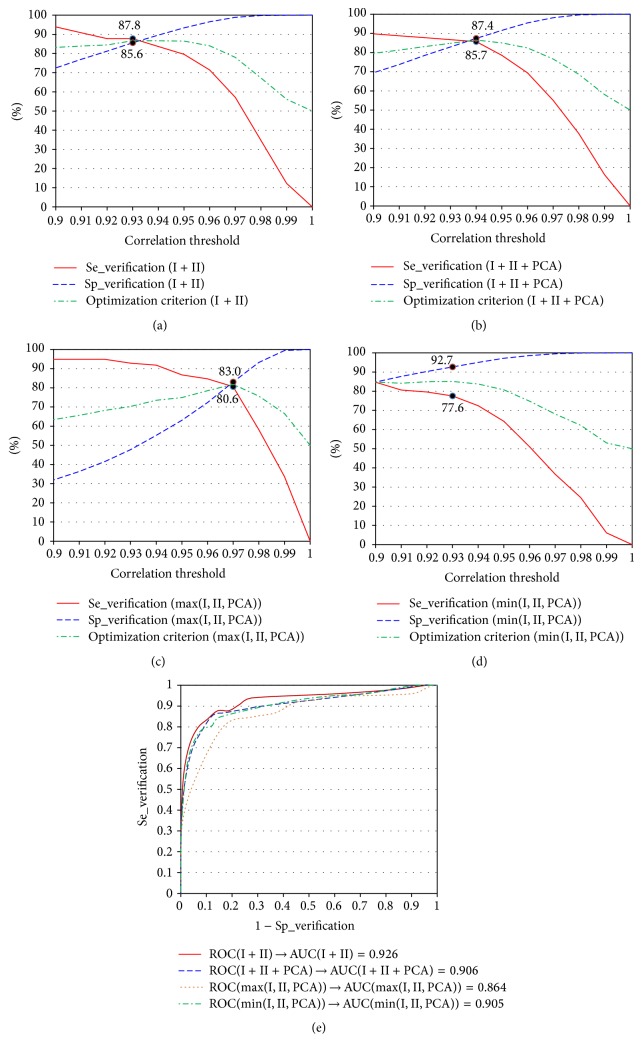
Influence of the threshold for (*r*_I_ + *r*_II_)/2, (*r*_I_ + *r*_II_ + *r*_PCA_)/3, max(*r*_I_, *r*_II_, *r*_PCA_), and min(*r*_I_, *r*_II_, *r*_PCA_) on the person verification accuracy: (a) Se_verification (I + II) = *f*((*r*_I_ + *r*_II_)/2); Sp_verification (I + II) = *f*((*r*_I_ + *r*_II_)/2). (b) Se_verification (I + II + PCA) = *f*((*r*_I_ + *r*_II_ + *r*_PCA_)/3); Sp_verification (I + II + PCA) = *f*((*r*_I_ + *r*_II_ + *r*_PCA_)/3). (c) Se_verification (max(*r*_I_, *r*_II_, *r*_PCA_)) = *f*(max(*r*_I_, *r*_II_, *r*_PCA_)); Sp_verification (max(*r*_I_, *r*_II_, *r*_PCA_)) = *f*(max(*r*_I_, *r*_II_, *r*_PCA_)). (d) Se_verification (min(*r*_I_, *r*_II_, *r*_PCA_)) = *f*(min(*r*_I_, *r*_II_, *r*_PCA_)); Sp_verification (min(*r*_I_, *r*_II_, *r*_PCA_)) = *f*(min(*r*_I_, *r*_II_, *r*_PCA_)). (e) ROC curves and calculated AUC for classification based on *r*_I_ + *r*_II_, *r*_I_ + *r*_II_ + *r*_PCA_, min(*r*_I_, *r*_II_, *r*_PCA_), and max(*r*_I_, *r*_II_, *r*_PCA_). The solid circles mark the optimal solutions.

Threshold values for *r*_I_, *r*_II_, *r*_PCA_, (*r*_I_ + *r*_II_)/2, (*r*_I_ + *r*_II_ + *r*_PCA_)/3, max(*r*_I_, *r*_II_, *r*_PCA_), and min(*r*_I_, *r*_II_, *r*_PCA_) are selected, based on analysis of the relation between their values and the verification accuracy (see Figures 1(a)–1(c) and 2(a)–2(d)). Using max(Se_verification + Sp_verification) as an optimization criterion, we selected threshold values for *r*_I_ (0.96), *r*_II_ (0.92), *r*_PCA_ (0.95), (*r*_I_ + *r*_II_)/2 (0.93), (*r*_I_ + *r*_II_ + *r*_PCA_)/3 (0.94), max(*r*_I_, *r*_II_, *r*_PCA_) (0.97), and min(*r*_I_, *r*_II_, *r*_PCA_) (0.93). The achieved accuracy indices are presented in Table 1. Receiver operating characteristic (ROC) curves are built and the area under the curve (AUC) is calculated for *r*_I_, *r*_II_, *r*_PCA_, (*r*_I_ + *r*_II_)/2, (*r*_I_ + *r*_II_ + *r*_PCA_)/3, max(*r*_I_, *r*_II_, *r*_PCA_), and min(*r*_I_, *r*_II_, *r*_PCA_). AUC could be used for scoring the potential for person verification of different models.

*(2) Person Identification*. The one-to-many scenario is applicable to a specific group of persons. The ECG in *T*_2_ of the subject under identity examination is compared to all previously recorded ECG in *T*_1_ and the maximal correlation of this comparison detects the identity to the tested subject. The identification accuracy (AccID) is calculated as the percentage of subjects for whom ECG in *T*_2_ is maximally correlated with their own ECG in *T*_1_.

## 3. Results

The Se_verification, Sp_verification, and the value of the optimization criterion achieved for the training database with the selected thresholds for the *r*_I_, *r*_II_, *r*_PCA_, and their combinations are presented in Table 1. The accuracy for person identification, calculated as the percentage of subjects, whose ECG in *T*_2_ is maximally correlated with their own ECG in *T*_1_ is also presented in Table 1.

**Figure 3:**
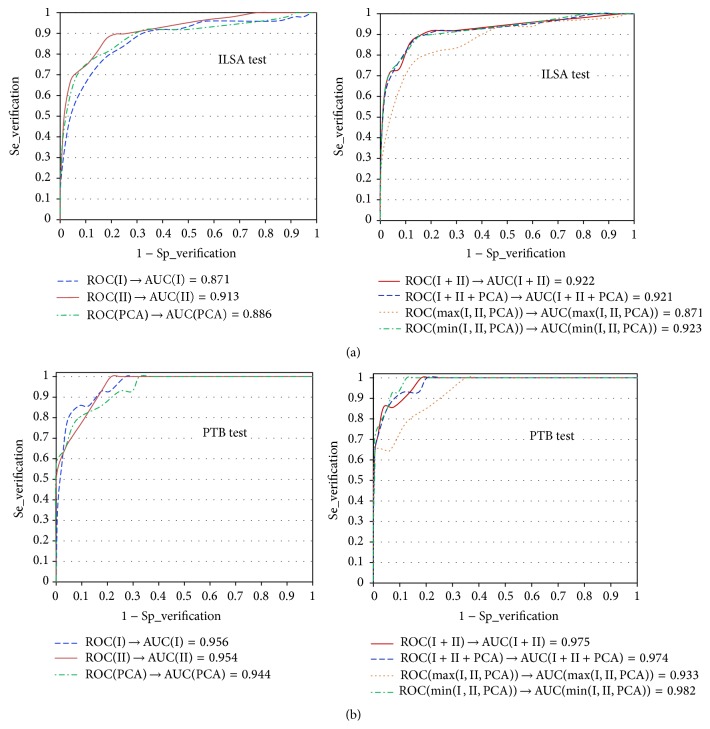
ROC curves and calculated AUCs for the test datasets: (a) ILSA test dataset (healthy subjects), (b) PTB healthy controls.

The proposed method for person verification/identification was independently tested over the two test databases. For the verification task, the correlation coefficients threshold values observed over the training database were applied. Aiming to obtain comparable results to the one reported in literature and to provide an unbiased basis for assessment of the influence of the personal health status on the verification/identification accuracy, we performed 2 types of tests as follows:

- Tests include only the healthy subjects: the results are presented in Table 2 for ILSA test dataset (49 persons) and Table 4 for PTB test dataset (14 persons).  Figure 3 illustrates the ROC curves with the respective AUCs that prove the similar behavior of the designed person validation method over the training and test ILSA datasets.
- Tests include all patients from the ILSA test dataset (442 persons): the results for verification and identification accuracy are presented in Table 3, considering the three groups of patients: healthy, cardiac, and hypertension ones.

**Figure 4:**
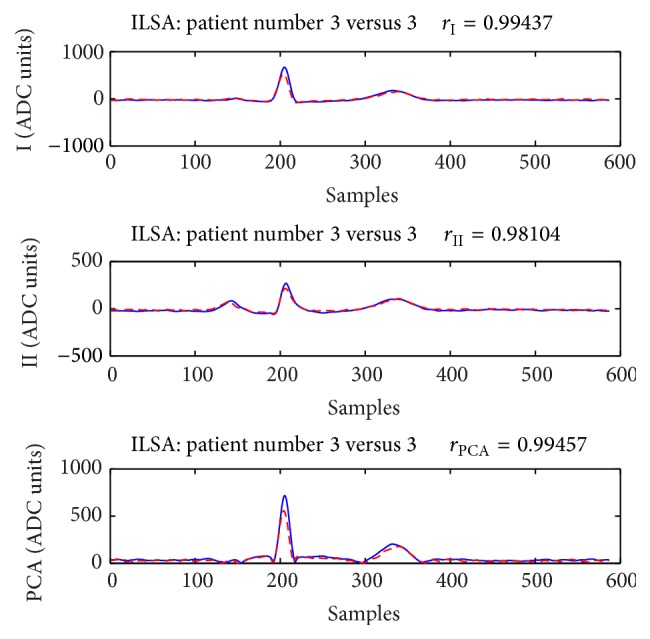
Subject 3 from the ILSA database. The strong correlation (>0.98) between the P-QRS-T waveforms of this person of the training set in *T*_1_ (blue solid line) and *T*_2_ (red dashed line) assures both correct verification and correct identification. The time interval between the recordings of ECG in *T*_1_ and *T*_2_ is about 5 years.

**Figure 5:**
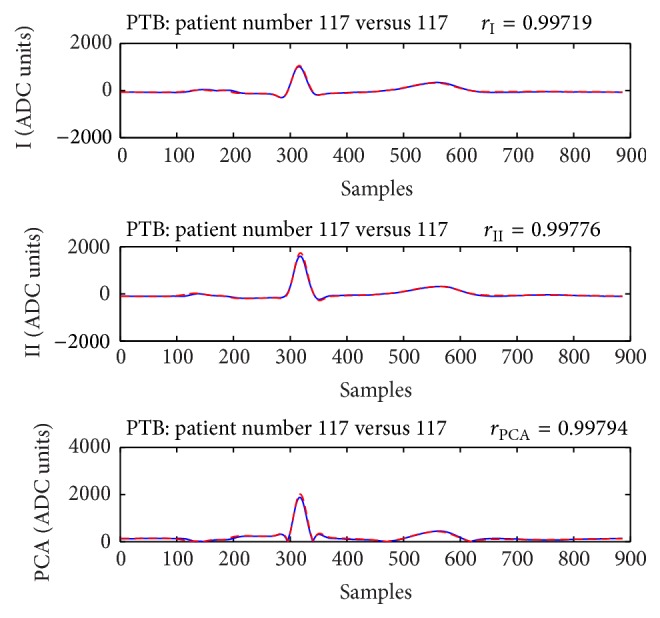
Subject 117 from the PTB database. The strong correlation (>0.99) between the P-QRS-T waveforms of this person in *T*_1_ (blue solid line) and *T*_2_ (red dashed line) assures both correct verification and correct identification. The time interval between the recordings of ECG in *T*_1_ and *T*_2_ is less than 24 hours.

Examples that show cases of both correct person verification and identification are presented in Figure 4 for the training database and Figure 5 for the publicly available PTB test dataset. Despite the strong correlations observed in both examples, it is visible that there is a complete matching between the waveforms of the ECGs in Figure 5, while the ECGs in Figure 4 show slight changes mainly in the QRS amplitudes.

**Figure 6:**
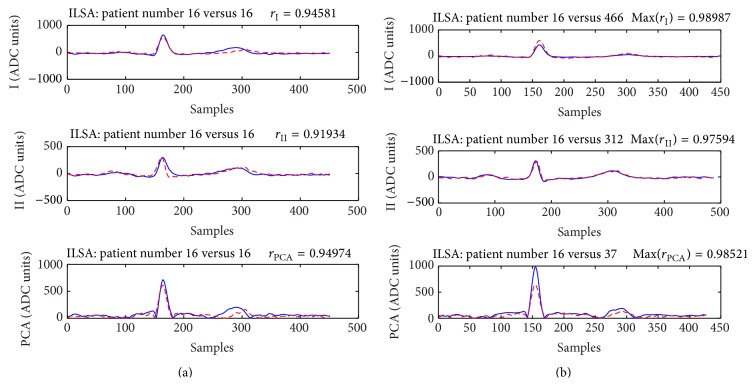
Subject 16 from the test part of ILSA database. (a) The assessed correlation coefficients *r*_I_ < 0.946, *r*_II_ = 0.919, and *r*_PCA_ < 0.949 are below the preset threshold values (0.96, 0.92, and 0.95, resp.) and the identity of this person will not be verified neither by means of *r*_I_, *r*_II_, and *r*_PCA_ nor by applying any of their combinations. (b) This subject will be identified as subjects 466 (Max(*r*_I_) ~ 0.99, max(*r*_I_, *r*_II_, *r*_PCA_) = 0.99); 312 (Max(*r*_II_) ~ 0.98), 37 (Max(*r*_PCA_) ~ 0.99); 134 ((*r*_I_ + *r*_II_)/2 = 0.95, (*r*_I_ + *r*_II_ + *r*_PCA_)/3 = 0.96); or 439 (min(*r*_I_, *r*_II_, *r*_PCA_) = 0.94). When the patient's ECG is compared to his own previously recorded ECG (*r*_I_ + *r*_II_)/2 = 0.93, (*r*_I_ + *r*_II_ + *r*_PCA_)/3 = 0.94, max(*r*_I_, *r*_II_, *r*_PCA_) = 0.95, and min(*r*_I_, *r*_II_, *r*_PCA_) = 0.92.

**Figure 7:**
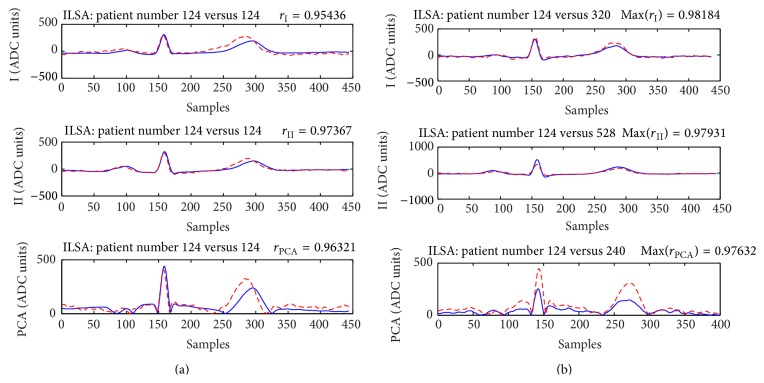
Subject 124 from the training part of ILSA database. Considering the independent correlation coefficients *r*_I_, *r*_II_, and *r*_PCA_, this subject will be identified either as subjects 320 (Max(*r*_I_) = 0.98, max(*r*_I_, *r*_II_, *r*_PCA_) = 0.98), 528 (Max(*r*_II_) = 0.979), 240 (*r*_PCA_ = 0.98) or as subject 255 (min(*r*_I_, *r*_II_, *r*_PCA_) = 0.96). However, the combined correlation coefficients (*r*_I_ + *r*_II_)/2, (*r*_I_ + *r*_II_ + *r*_PCA_)/3 both have maximal values of 0.96 when the ECG in *T*_2_ of this person is compared to his own ECG in *T*_1_.

**Figure 8:**
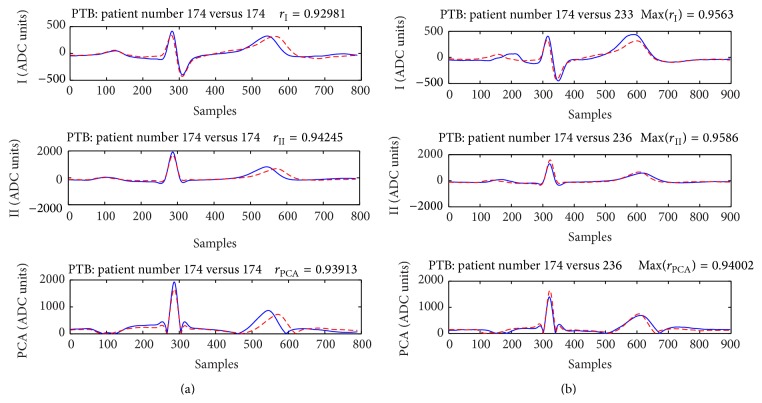
Subject 174 from the PTB database-time interval between the recordings of ECG in *T*_1_ and *T*_2_ is 59 days. (a) The assessed correlation coefficients *r*_I_ = 0.93 and *r*_PCA_ = 0.94 are below the preset threshold values (0.96 and 0.95, resp.) and the identity of this person will not be verified. (b) This subject will be identified as 233 according to Max(*r*_I_) ~ 0.96, (*r*_I_ + *r*_II_)/2 = 0.95, (*r*_I_ + *r*_II_ + *r*_PCA_)/3 = 0.95, min(*r*_I_, *r*_II_, *r*_PCA_) = 0.94, or as 236 if Max(*r*_II_) ~ 0.96, Max(*r*_PCA_) ~ 0.97, and max(*r*_I_, *r*_II_, *r*_PCA_) = 0.97 are considered. When the patient's ECG is compared to his own previously recorded ECG (*r*_I_ + *r*_II_)/2 = 0.94, (*r*_I_ + *r*_II_ + *r*_PCA_)/3 = 0.94, max(*r*_I_, *r*_II_, *r*_PCA_) = 0.94, and min(*r*_I_, *r*_II_, *r*_PCA_) = 0.93.

Figures 6 and 8 present cases for which neither the verification nor the identification will be successful. The example in Figure 7 illustrates a case, which will be correctly verified if *r*_II_, *r*_PCA_, or the combined correlation coefficients are used and erroneously rejected if *r*_I_ < 0.96 is applied for verification. The only chance this person to be correctly identified is the application of one of the combined correlation coefficients (*r*_I_ + *r*_II_)/2 and (*r*_I_ + *r*_II_ + *r*_PCA_)/3.

## 4. Discussion

This paper presents a method for person verification and identification based on cross-correlation over ECG signals. Aiming to assure a convenient and a comfortable acquisition procedure for the tested person, the proposed algorithm uses only the independent limb leads I, II.

The person verification is performed by comparison between the ECG of the person who pretends for certain identity and a previously recorded ECG of subject with the tested identity, using the maximal cross-correlation as an estimator for their similarity. The curves in Figures 1(a)–1(c) and 2(a)–2(d) provide the opportunity to select threshold values for *r*_I_, *r*_II_, *r*_PCA_, (*r*_I_ + *r*_II_)/2, (*r*_I_ + *r*_II_ + *r*_PCA_)/3, max(*r*_I_, *r*_II_, *r*_PCA_), and min(*r*_I_, *r*_II_, *r*_PCA_) depending on the application. Lower thresholds are suitable when low rejection rate (high sensitivity) is required at the expense of increased erroneous verifications. On the other side, the higher threshold values guarantee high security combined with higher rejection rate. Depending on the exact task that has to be solved, one can decide threshold values of the applied correlation coefficients. The ROC curves and AUCs presented in Figures 1(d) and 2(e) illustrate the behavior of the models over the train dataset. According to [22], AUC higher than 0.9 is an approximate indication of an excellent classifier.

The person identification is performed by computing the correlation between the ECG of the subject under examination and a previously collected ECG database. The tested person is identified according to the maximal correlation to a subject in the database.

Considering the accuracy results (Tables 1, 2, and 3) over the training and test ILSA database and the AUCs (Figures 1(d) and 3), *r*_II_ seems more reliable for person verification than *r*_I_. However, this observation is not confirmed by the results over the test PTB database (Table 4, Sp_verification < 70%). Although the first PCA component presents a combination between leads I and II, *r*_PCA_ do not lead to verification accuracy increase neither for the training nor for the test databases. Generally, the best verification/identification accuracy is achieved with the combined correlation coefficients (*r*_I_ + *r*_II_)/2, (*r*_I_ + *r*_II_ + *r*_PCA_)/3 that also present AUCs higher than 0.9 (Figure 2(e) for training, Figure 3 for testing). This is in concord with the results of Poree et al. [7], who reported accuracy increase when more ECG leads are involved in the analysis.

Considering the verification/identification of healthy persons, the following observations can be pointed out:

(i) There is comparable verification accuracy for both training and test part of ILSA database (Table 1 versus Table 2, AUCs in Figures 1(d) and 2(e) versus Figure 3).
(ii) The identification accuracy is higher in the healthy ECG-ILSA test set (*N* = 49Table 2) than in the learning set (*N* = 98, Table 1).
(iii) There is lower identification accuracy for the test ILSA dataset when all ECGs in the ECG-ILSA test set (*N* = 442) are used as database for comparison (Table 3 versus Tables 1 and 2).
(iv) There is higher verification/identification accuracy for the test PTB dataset compared to the ECG-ILSA training and test sets.

These observations can be motivated by the following remarks:

(i) The better verification accuracy for the test PTB dataset can be explained by the shorter temporal interval between the two acquisition times *T*_1_ and *T*_2_, compared to the temporal interval of 5 years in the ECG_ILSA database which is in concord with the observations in [14]. In fact the 14 subjects are characterized by
  (a) (*T*_2_ − *T*_1_) < 24 hours in 7 cases,
  (b) (*T*_2_ − *T*_1_) < 1 month in 2 patients,
  (c) (*T*_2_ − *T*_1_) > 1 and < 3 months in 2 patients,
  (d) (*T*_2_ − *T*_1_) > 6 months for 3 patients.
(ii) The identification accuracy is influenced by the number of records in the testing set, and, consequently with reduced number of patients, it is possible to obtain higher values. In fact, the PTB database with only 14 records produces better identification accuracy (92.9% with (*r*_I_ + *r*_II_)/2 in Table 4) if compared with the test set of 49 healthy people of the ECG-ILSA database (Table 2) or if compared with all 442 subjects of the test set (Table 3). This behavior is in agreement with the study of Zokaee and Faez [10] where the increasing from 10 to 50 of the number of ECGs in the test dataset produced decreasing of the accuracy of about 10% (from 98.6% to 89%).

In literature there are only few studies [5] which consider the ECG biometric recognition in the presence of cardiac irregularity conditions, although they were performed mainly with single day sessions. Consequently, the present work represents a significant test on the influence of the personal health status for the verification/identification accuracy in the presence of long term interval recordings.

Our observations for cardiac and hypertension persons are as follows.

Considering the (*r*_I_ + *r*_II_)/2 classification method, the validation procedure on the entire test set of 442 healthy and nonhealthy patients, some observations can be performed as follows:

- The hypertension group (Table 3) shows lower Se_verification (82.6%) and AccID (53.7%) compared to the healthy and cardiac groups (resp., 89.8%, 86.3% and 59.2%, 60.6%).
- The cardiac group shows a slight improvement in AccID (60.6%) and in Sp_verification (91.8%) in comparison with the healthy group (59.2% and 86.6%).

These results show the robustness of the proposed classification method for person identification and verification, although the obtained results are probably not effective for real applications. It is interesting to consider the classification of some examples with or without problematic identification/verification.

The example in Figure 5 shows strong correlation between 2 ECG recordings of one subject from the test database that lead to unconditional correct person verification and identification. It should be mentioned, however, that both ECGs are recorded within time interval less than 24 hours. High values of the correlation coefficients are also observed for ECG recordings with temporal interval of several years (see the example in Figure 4); however, for such cases the expected ECG changes are visible.

On the other pole, the examples in Figures 6 and 8 present aligned P-QRS-T segments of subjects, for whom most of the correlation coefficients are below the thresholds for person verification. Moreover, the ECGs of these people are more correlated with ECGs of other members in the datasets used for comparison which leads to incorrect identification.

The example in Figure 7 proves the advantages of the combined correlation coefficients (*r*_I_ + *r*_II_)/2 and (*r*_I_ + *r*_II_ + *r*_PCA_)/3 that prevent erroneous identifications when strong correlation with a wrong ECG from the database for comparison is observed only in lead I, lead II, or the first PCA component.

Considering the differences in the verification/identification accuracy over the training and test databases, as well as the presented examples for correct and erroneous verification, we can conclude that the changes appearing in ECG with time could affect the accuracy of person verification/identification. This is also confirmed by the comparison between our results on healthy persons and the results reported in literature (see Table 5). It is obvious that the studies using only close in time ECG recordings [3, 10, 11] report higher identification and/or verification performances. The direct comparison to studies that use the healthy controls in PTB database is not possible, since the authors have applied their methods on part of the healthy persons (12 or 13 from all 14 patients) and have mixed them with cases that do not have separated in time ECGs. Nevertheless, our results could be positively compared with studies for the identification task which consider ECG datasets with comparable size and similar temporal interval between the 1st and the 2nd recording [1, 5, 7, 10].

The ECG variability within small time interval could be due to electrode position variation between the two recordings. Even a meal can cause considerable ECG changes, both in healthy people and in cardiac patients. ECG recordings acquired one or more years apart show larger intraindividual variability. Sources such as age, weight, and heart position then come into play, in addition to the sources already having effect on smaller time scales. The influences of different factors on the intraindividual ECG variability are summarized by Schijvenaars [24]. In healthy people, the most prominent changes after a standard meal are an increase of heart rate, a decrease of T-wave amplitude and QT interval, and small left axis shifts of the QRS and T-axes. The influences of age, weight, and heart position are often interdependent; the heart position becomes more horizontal when one gains weight, people generally gain some weight as years pass, and so forth. The general trend is a decrease in amplitudes and a left axis shift in frontal QRS axis with increasing age or weight. The general age trends found among adults are decrease of precordial amplitudes (QRS spatial magnitude decreases with approximately 8% per decade), a leftward shift of the frontal plane axis (approximately 10° per decade), and a more anterior axis in the horizontal plane. Interval durations' increase for PR and QT interval and decrease in QRS duration are also observed.

Considering the above mentioned sources of ECG variability, as well as our observations over the training and test databases, we conclude the following.

- Aiming at higher identification accuracy, the database for comparison (TrainSet1, TestSet1) should be kept as small as possible for the particular application and should be updated with actual ECG recordings.
- Aiming at higher verification accuracy, the ECG recordings in the database for comparison should be updated on a regular basis (as short as possible, e.g., every time when the person passes through border control). The new ECG could replace the old one, after verification, or could be added to a personal folder with a reasonable size. This would guarantee higher values of Se_verification and would provide the opportunity to increase the threshold values for the correlation coefficients which in turn would increase Sp_verification.

## 5. Conclusions

This paper studies the reliability of the ECG signal for person verification/identification. The population based ECG-ILSA database of 540 patients (147 healthy subjects, 175 patients with cardiac diseases, and 218 with hypertension) has been considered for the validation procedure. For a more robust validation procedure, considering the temporal variability of ECG signal and/or modifications in the pathologies, the learning phase was performed only in the healthy group, and the testing procedures have been performed also with nonhealthy patients. The proposed method relies on assessment of correlation coefficients as well as their linear and nonlinear combinations and provides 100% verification sensitivity combined with 18.1% erroneous verification rate in the PTB database, a widely used test set in literature, with a relatively short temporal interval of ECG acquisition and a limited number of healthy subjects (*N* = 14). This dataset produces an identification accuracy of 92.9%. The test set of healthy subjects in the ECG-ILSA database (*N* = 49) produces a lower verification sensitivity (89.8%) and identification accuracy (77.6%) but a better erroneous verification rate (16.1%). For cardiac and hypertension patients we observe decreased sensitivity and increased specificity for verification. Considering the identification task, our conclusions are that the accuracy depends generally on the size of the database for comparison, but not on the person's health status.

Although the ECG is considered to be strongly individual biometric feature, this study shows that there are some changes over time that could prevent correct individual verification, and two healthy persons could have similar ECGs that could lead to incorrect identification. This poses requirements towards the database stored for comparison, such as size of the database and maximal time interval between the tested ECG and the ECG stored in this database. Despite these limitations, the ECG has the indisputable advantage to be not susceptible to falsification. It seems to be a reliable biometric characteristic for specific access control applications, which operate with smaller databases for comparison.

Although there are several limitations in this methodology, which can prevent its use in real practice, it could be possible to overcome the drawbacks with the inclusion of some demographic/personal information in the classification process for obtaining “certain” identification. Moreover, this study proves the potential of ECG application for increasing the reliability of person verification and identification based on biometrical information from other sources.

## Figures and Tables

**Table 1:** Verification/identification accuracy calculated over the training database: selected threshold value (VerTHR), sensitivity, specificity, and value of the optimization criterion for person verification; accuracy for person identification (AccID), *N* = 98.

	VerTHR	Se_verification	Sp_verification	Opt. criterion	AccID
*r*_I_	0.96	78.6% (77/98)	77% (7320/9506)	77.8%	53.1% (52/98)
*r*_II_	0.92	83.7% (82/98)	85.7% (8147/9506)	84.7%	59.2% (58/98)
*r*_PCA_	0.95	79.6% (78/98)	76.6% (7282/9506)	78.1%	52% (51/98)
(*r*_I_ + *r*_II_)/2	0.93	**87.8%** (86/98)	**86.6%** (8232/9506)	**86.7%**	**69.4%** (68/98)
(*r*_I_ + *r*_II_ + *r*_PCA_)/3	0.94	**85.7%** (84/98)	**87.4%** (8308/9506)	**86.6%**	**71.4%** (70/98)
max⁡(*r*_I_, *r*_II_, *r*_PCA_)	0.97	80.6% (79/98)	83.0% (7890/9506)	81.8%	59.2% (58/98)
min⁡(*r*_I_, *r*_II_, *r*_PCA_)	0.93	77.6% (76/98)	92.7% (8812/9506)	85.1%	65.3% (64/98)

**Table 2:** Sensitivity and specificity for person verification and accuracy for person identification obtained for the healthy subset in the test ILSA dataset (*N* = 49 subjects).

	Se_verification	Sp_verification	AccID
*r*_I_	77.6% (38/49)	83% (1912/2304)	63.3% (31/49)
*r*_II_	87.8% (43/49)	81.7% (1882/2304)	61.2% (30/49)
*r*_PCA_	81.6% (40/49)	80.7% (1859/2304)	59.2% (29/49)
(*r*_I_ + *r*_II_)/2	**89.8%** (44/49)	**83.9%** (1933/2304)	**77.6%** (38/49)
(*r*_I_ + *r*_II_ + *r*_PCA_)/3	**87.8%** (43/49)	**86.6%** (1995/2304)	**75.5%** (37/49)
max⁡(*r*_I_, *r*_II_, *r*_PCA_)	75.5% (37/49)	87.5% (2016/2304)	65.3% (32/49)
min⁡(*r*_I_, *r*_II_, *r*_PCA_)	79.6% (39/49)	90.6% (2087/2304)	75.5% (37/49)

**Table 3:** Sensitivity and specificity for person verification, together with the accuracy for person identification, obtained for the entire test ILSA dataset (*N* = 442 subjects), considering the health status of the tested subjects.

	Se_verification (%)			Sp_verification (%)			AccID (%)		
	Healthy	Card	Hypt	Healthy	Card	Hypt	Healthy	Card	Hypt
*r*_I_	77.6%	74.9%	76.1%	82.8%	87.9%	81.4%	38.8%	46.3%	46.3%
*r*_II_	87.8%	77.7%	78.9%	85.4%	90.8%	86.7%	42.9%	52.0%	44.5%
*r*_PCA_	81.6%	80.0%	79.4%	81.6%	85.0%	78.9%	42.9%	48.0%	41.3%
(*r*_I_ + *r*_II_)/2	**89.8%**	**86.3%**	**82.6%**	**86.6%**	**91.8%**	**87.0%**	59.2%	60.6%	53.7%
(*r*_I_ + *r*_II_ + *r*_PCA_)/3	**87.8%**	78.3%	79.8%	**88.9%**	**92.9%**	88.4%	**61.2%**	**62.3% **	**54.6%**
max⁡(*r*_I_, *r*_II_, *r*_PCA_)	75.5%	73.1%	74.8%	87.4%	90.9%	85.8%	42.9%	57.7%	46.8%
min⁡(*r*_I_, *r*_II_, *r*_PCA_)	79.6%	69.7%	71.6%	92.6%	95.6%	92.9%	59.2%	54.9%	49.5%

**Table 4:** Sensitivity and specificity for person verification and accuracy for person identification over the test PTB dataset (*N* = 14 subjects).

	Se_verification	Sp_verification	AccID
*r*_I_	85.7% (12/14)	91.2% (166/182)	92.9% (13/14)
*r*_II_	100% (14/14)	69.8% (127/182)	92.9% (13/14)
*r*_PCA_	92.9% (13/14)	75.3% (137/182)	78.6% (11/14)
(*r*_I_ + *r*_II_)/2	**100%** (14/14)	**81.9%** (149/182)	**92.9%** (13/14)
(*r*_I_ + *r*_II_ + *r*_PCA_)/3	**92.9%** (13/14)	**83.0%** (151/182)	**85.7%** (12/14)
max⁡(*r*_I_, *r*_II_, *r*_PCA_)	78.6% (11/14)	87.4% (159/182)	78.6% (11/14)
min⁡(*r*_I_, *r*_II_, *r*_PCA_)	92.9% (13/14)	90.7% (165/182)	92.9% (13/14)

**Table 5:** Comparison between verification/identification accuracy achieved by the proposed method over the test dataset and the results reported by other authors with different databases used (db). The number of ECG recordings per patient (1 rpp for one and mrpp for more) and the acquisition interval (acq_int) on the same patient are reported.

Method	Database	Accuracy
Agrafioti and Hatzinakos, 2009 [5]	MIT-BIH normal sinus MIT-BIH arrhythmia 1 rpp PTB db, 13 healthy subjects mrpp	AccID = 96.2% Sp_ver = 99% Se_ver = 87%

Israel et al., 2005 [3]	Own db: 29 subjects close in time recordings	AccID = 100%

Lourenço et al., 2011 [13]	Own db: 16 subjects close in time recordings	AccID = 94.3% Se = Sp = 87%

Sidek et al., 2012 [11]	Own db: 30 healthy subj., close in time recordings	AccID = 96.1%

Wang et al., 2008 [4]	MIT-BIH normal sinus 1 rpp PTB db, 13 healthy subj. mrpp	AccID = 100%

Zhao et al., 2013 [1]	MIT-BIH ST change db, long-term ST db 1 rpp; PTB db, 12 healthy subj. mrpp	AccID (tot) = 95.6% AccID (PTB) = 96%

Zokaee and Faez, 2012 [10]	MIT-BIH db 1 rpp Own Holter, 50 subjects 1 rpp	AccID = 100% AccID = 89%

Poree et al., 2011 [7]	Own db: 11 subjects, mrpp, acq_int = 16 months	AccID = 91.4%

Lee et al., 2012 [23]	Own db: 10 subjects, ~100 rpp within 3-month period	AccID = 99.5%

Wübbeler et al., 2007 [8]	db from 74 subjects, mrpp, acq_int = 16 months	AccID = 98.1% Se = Sp = 97.2%

Our method (based on assessment of (*r*_I_ + *r*_II_)/2)	Test ILSA db, 49 healthy subjects mrpp	AccID = 77.6% Se_ver = 89.8% Sp_ver = 83.9%
	Test PTB db, 14 healthy subjects mrpp	AccID = 92.9% Se_ver = 100% Sp_ver = 81.9%
